# The Relevance of Short-Range Fibers to Cognitive Efficiency and Brain Activation in Aging and Dementia

**DOI:** 10.1371/journal.pone.0090307

**Published:** 2014-04-02

**Authors:** Junling Gao, Raymond T. F. Cheung, Ying-Shing Chan, Leung-Wing Chu, Henry K. F. Mak, Tatia M. C. Lee

**Affiliations:** 1 Department of Medicine, Li Ka Shing Faculty of Medicine, The University of Hong Kong, Hong Kong, P. R. China; 2 Alzheimer's Disease Research Network, Strategic Research Theme of Healthy Aging, The University of Hong Kong, Hong Kong, P. R. China; 3 Research Center of Heart, Brain, Hormone and Healthy Aging, Li Ka Shing Faculty of Medicine, The University of Hong Kong, Hong Kong, P. R. China; 4 Department of Physiology, Li Ka Shing Faculty of Medicine, The University of Hong Kong, Hong Kong, P. R. China; 5 Department of Radiology, Li Ka Shing Faculty of Medicine, The University of Hong Kong, Hong Kong, P. R. China; 6 Laboratory of Cognitive Affective Neuroscience, Faculty of Social science, The University of Hong Kong, Hong Kong, P. R. China; 7 The State Key Laboratory of Brain and Cognitive Sciences, The University of Hong Kong, Hong Kong, P. R. China; 8 Laboratory of Neuropsychology, Faculty of Social Science, The University of Hong Kong, Hong Kong, P. R. China; Wake Forest School of Medicine, United States of America

## Abstract

The integrity of structural connectivity in a functional brain network supports the efficiency of neural processing within relevant brain regions. This study aimed to quantitatively investigate the short- and long-range fibers, and their differential roles in the lower cognitive efficiency in aging and dementia. Three groups of healthy young, healthy older adults and patients with Alzheimer's disease (AD) participated in this combined functional magnetic resonance imaging (fMRI) and diffusion tensor imaging (DTI) study on prospective memory (PM). Short- and long-range fiber tracts within the PM task engaged brain networks were generated. The correlation between the fMRI signal change, PM performance and the DTI characters were calculated. FMRI results showed that the PM-specific frontal activations in three groups were distributed hierarchically along the rostrocaudal axis in the frontal lobe. In an overall PM condition generally activated brain network among the three groups, tractography was used to generate the short-range fibers, and they were found impaired in both healthy older adults and AD patients. However, the long-range fiber tracts were only impaired in AD. Additionally, the mean diffusivity (MD) of short-range but not long-range fibers was positively correlated with fMRI signal change and negatively correlated with the efficiency of PM performance. This study suggests that the disintegrity of short-range fibers may contribute more to the lower cognitive efficiency and higher compensatory brain activation in healthy older adults and more in AD patients.

## Introduction

Studies on brain connectivity have advanced considerably and helped to understand cognition efficiency and relevant impairment in neurobiological diseases. Numerous functional connectivity studies have found that the brain has both high local clustering coefficiency and optimal global integration, similar to a small-world network [1], [2]. Only a few studies have explored the relationships of structural connectivity and functional activity [3]. A developmental study found that white matter connectivity supports brain-wide coherence and synchrony [3]. Another study showed decoupling between functional and structural connectivity in Schizophrenia patients [4]. These studies indicated that combining functional and structural neuroimaging studies can more comprehensively assess the altered brain connectivity in different clinical conditions [5].

Parallel with the division of clustering and global integration in functional connectivity study, the structural connectivity can further be divided into regional short-range fibers that specified for local connectivity and long-range fiber for more global connectivity [6]–[8]. For example, short-range fibers mainly include the local associative fibers (U-shaped) connecting intraterritorial connections and neighborhood association fibers connecting adjacent areas [9]. Given that the intrinsic functional connectivity is disrupted in dementia [10], it is plausible that the impairment of short- and long-range fibers may play important roles in the lower cognitive efficiency in aging and dementia.

The structural and functional interaction between brain regions gives rise to cognition, and the quality of interaction change with normal and pathological aging [5]. The complexity of brain connectivity makes it susceptible to the effect of aging and diseases, such as the Alzheimer's disease (AD), the most common dementia in clinics [11]. Both structural connectivity and functional connectivity are impaired in advanced aging [12], [13]. For example, Voineskos et al. found that the microstructural integrity of major fasciculus continues to decline during the lifespan from the age 20 s to 80 s, and specific white matter degeneration may account for different age-related cognitive decline [14]. The impairments are more advanced in AD, which is regarded as a disconnection syndrome [15], [16]. DTI may also help to assess the modifying effect of pharmacological interventions on AD [17], [18]. A multicenter DTI study reported that cingulate bundle is impaired in probable AD when compared to healthy controls, using automated tractography [19].

Nonetheless, few multi-modal neuroimaging studies have investigated the aging process, including both normal and pathological aging. The different roles of short- and long-range fibers in these aging processes were never explored. To solve the aforementioned problems, we chose to examine an important cognitive function, the prospective memory (PM). PM represents the ability to remember something to be executed in the future, e.g, to see a doctor or to take medicine before sleep [20]. There has been a surge of interest in PM research, since PM performance is related to executive function [21], and critical to the independence in daily living [22], especially in the vulnerable populations including old adults and those with AD [23]. Some studies have shown that PM is impaired in older adults [23], [24], whereas others found little age-related decline in PM [25], [26]. This inconsistence may be caused by different PM paradigms or different strategies adopted by the individuals [26]. On the other hand, there is consistent evidence of PM deficit in AD patients, and PM could be more impaired than retrospective memory [23], [27].

Previous neuroimaging studies on PM using only young subjects have shown that a network of brain regions is involved in PM task performance [28]–[30], including the frontal-parietal regions, the precuneus, the supplementary motor area, etc. [28]. Although a number of behavioral studies have shown PM impairment in aging and AD [31]–[34], neuroimaging study on the old adults and AD patients to explore the neural correlates of PM impairment is rare [35], [36], given the obvious difficulty for them to accomplish the task in the scanner. A better understanding on neural correlates in PM task may help to design appropriate neurorehabilitation strategies for the vulnerable population [37].

This neuroimaging study aimed to investigate the brain regions supporting PM function with a broader range of population including healthy young adults, healthy older adults and AD patients as a continuum. This design would enable greater power to explore the correlation between structural connectivity with cognitive function. The hypothesis was that the integrity of structural connectivity among the PM activated brain regions may be impaired in older adults, and this impairment could be correlated with their lower efficiency in PM task performance. Inspired by the division of local clustering and global integrity, we would separately explore the integrity of short- and long-range fibers in structural connectivity analysis, as they might contribute differentially to lower cognitive efficiency in aging and AD.

## Methods

### Subjects

The research protocol was approved by Institutional Review Board in Queen Mary Hospital, and it was conducted in accordance with the Declaration of Helsinki. A written consent was obtained from each subject. We recruited 13 healthy young adults, 13 healthy older adults and 17 patients with mild AD. AD was diagnosed following the criteria of National Institute of Neurological Disorders and Stroke/Alzheimer's Disease and Related Disorders Association (NINDS-ADRDA) [38]. Subjects with a history of stroke, head injury, depression or any other major physical or affective disease that would affect cognitive function were excluded [39].

Several screening tests were administered upon subject recruitment: the Ishihara test [40], Edinburgh handedness inventory [41], Hachinski ischemic scale [42] and depression scales in order to exclude subjects with color blindness, left-handedness, vascular dementia and depression, respectively. AD patients and the healthy older adults were assessed additionally with the mini mental state examination (MMSE) [43], and clinical dementia rating scale (CDR) [44]. Other cognitive tests, such as digit span, digit-symbol modalities test, verbal fluency and delayed picture recognition were also administered. Participants' demographics and screening assessments are summarized in Table 1.

**Table 1:** Demographics of participants.

	Young(13)	Old(12)	AD(13)
**Age**, (yrs)	27.1±3.6	76.2±4.1*	76.7±2.4*
**Sex** (male/female)	4/7	7/5	8/5
**Education** (yrs)	16.2±3.3	4.4±2.6*	5.0±3.8*
**MMSE** (0 to 30)	N.A.	29.2±0.6	21.2±2.9#
**CDR** a	N.A.	0.00±0.00	0.74±0.19
**HIS** (0 to 18)	N.A.	2.5±1.9	3.2±2.0
**Depression scores** b	4.2±2.7	2.8±1.5	3.0±0.8
**Digital span** (forward)	10.7±2.0	7.8±1.9*	6.8±1.5*
**Digital span** (backward)	6.6±1.9	4.1±1.3*	3.1±2.1*
**Verbal fluency** (fruits/vegetables)	26.8±5.5	20.5±5.0*	9.8±3.5* #
**Verbal fluency** (animals)	25.0±8.0	19.5±6.6	9.7±3.6* #
**SDMT** (written)	57.7±8.4	27.8±11.8*	16.7±10.7* #
**SDMT** (spoken)	68.8±9.5	40.3±12.6*	23.0±11.0* #
**Picture recall** (delayed)	9.4±1.4	7.3±1.3*	3.9±1.5* #

The data are shown in Mean±SD. Numbers of subjects are shown in brackets. AD, patients with Alzheimer's disease; Old, healthy older adults; Young, young adults; MMSE, mini mental state examination; CDR, clinical dementia rating scale; HIS, Hachinski ischemic scale; SDMT, symbol digital modalities test; Picture recall: 5 minutes delayed recognition of 10 pictures.

a , 2 AD and 4 healthy older adults did not have CDR;

b , Depression score in young adults was derived from Beck Depression Inventory, and it was derived from Geriatric Depression Scale in the older adults and AD patients;

*, Group difference is significant when compared to healthy young adults, p<0.05;

# , Group difference is significant when compared to healthy older adults, p<0.05.

Half of the AD patients were regularly taking one cholinesterase inhibitor for treatment of dementia, but no vascular effective or cholinesterase drug was taken 4 hours before the MRI scanning. A T2-weighted MRI was done to identify hyperdensity for exclusion. Fazekas scale >1 was used to exclude participants with intensive white matter abnormalities [45]. As the performance within MRI became more difficult than that outside, 4 AD patients and 1 healthy older adult had either poor PM task performance or excessive movement. Their data were excluded from data analysis. Finally, the data of 13 AD patients, 12 healthy older adults and 13 young adults were analyzed and reported.

### Experimental design

**Figure 1:**
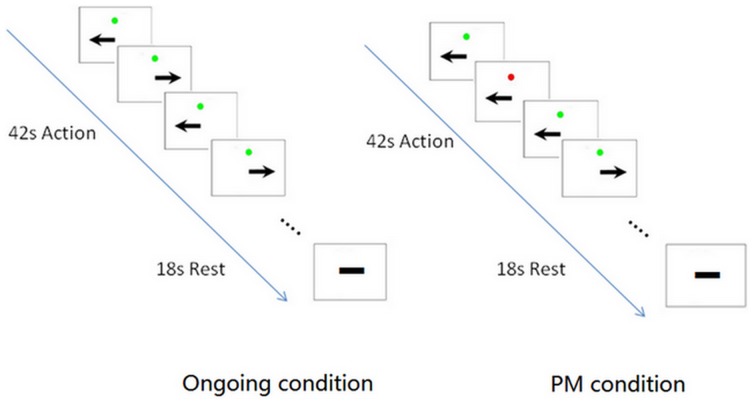
PM paradigm used in fMRI experiment. Arrow with a green-dot is the ongoing trial, arrow with a red-dot is the PM trial. The arrow could be either leftward or rightward. Ongoing trials appeared in both conditions, PM trials appeared only in PM condition. There was an instruction before each condition, then the action and rest blocks alternated four times in each condition.

The PM task was adapted from the arrow and color-bar PM task in a previous neuroimaging study on PM of young adults [46]. Revisions were made so that the healthy older adults and AD patients could successfully perform the task inside the MRI scanner. The PM task had two conditions: an ongoing condition as the baseline, and a PM condition interpolated with PM trials. The ongoing condition was always performed first and contained only ongoing trials, which had a green dot in the center of the screen together with a black arrowhead below (see Figure 1). The subjects were instructed to press the right index finger for a leftward arrow, and the right middle finger for a rightward arrow, as quickly and accurately as possible. In the separate PM condition, there were ongoing trails as well, but PM trials (22%) were interpolated with ongoing trials. In the PM trials, a red dot was in the center instead, and the subjects were instructed to only press the left index finger regardless of the arrowhead direction. This was to mimic the situation to ignore the ongoing task and shift to PM task in real life [24], [26]. Each trial would disappear immediately after a response (or the maximum allowed response time is 3000 ms). There was a fixation before each stimulus with a jittering interstimulus interval (ISI) of 800, 1000 or 1200 ms.

The timeline of block design was the same in both conditions. There was 12 seconds for instruction and preparation at the beginning, followed by 4 alternate blocks of 42-second active task and 18-second rest. The total scanning time was 252 seconds for each condition. The trial sequence was pseudo-randomized. That is, trials were randomized in each condition beforehand, while all the subjects performed the PM task with a same trial sequence. The ongoing condition was always before the PM condition. The two conditions were separately scanned in two consecutive sessions, and the first 7 trials of PM condition were ongoing trials in order to reduce continuous monitoring of PM trials [46]. All subjects had sufficient training to be familiar with PM performance outside and in the scanner. As AD patients had much difficulty to do PM task in the MRI scanner, they usually need longer practice before they could reach the criteria of at least 60% accuracy in the PM task.

### Neuroimaging protocols

Each subject lied supine in the scanner. Structural images were collected first so that the subjects became familiar with environment. During fMRI scanning, the stimuli were presented by an MRI-compatible projector using E-prime. The stimuli were centered on the screen with a visual angle of about 10 degrees. All subjects claimed to be able to view the stimuli clearly.

#### 3D anatomical T1 and T2 images acquisition

T1-weighted images were acquired on a 3.0T Philips MRI machine, using the following parameters: FoV = 256×150×240 mm, acquisition matrix = 256×256, TR = 15 ms, TE = 3.26 ms, flip angle = 25°, slice thickness = 1.5 mm, number of slices = 100, voxel resolution (x,y,z,) = 0.94×1×1.5 mm. The T1 image scan duration was 361 s. T2-weighted scan was acquired following a routine sequence used in clinics for abnormal hyperintensity.

#### Diffusion tensor imaging (DTI) data acquisition

Single shot echo-planar diffusion weighted imaging was collected with the following parameters: FoV = 224×140×224 mm, acquisition matrix = 112×112, TR = 9583 ms, TE = 62 ms, flip angle = 90°, slice thickness = 2 mm, number of slices = 70, voxel resolution (x,y,z,) = 2×2×2 mm. Diffusion sensitizing gradient (b = 800 s/mm2) was applied in 15 directions, one additional image was collected without diffusion gradient (b_0_ = 0 s/mm2). The DTI sequence was scanned twice and only the averaged DTI images were generated by the Philips MRI scanner. The DTI data scan duration was 358 s.

#### Functional MRI data acquisition

The fMRI images were obtained with gradient echo-planar imaging (EPI), with an 8 elements SENSE head coil reducing claustrophobic effects of scanner on the subjects. Other parameters were: FoV = 230×140×230 mm, acquisition matrix = 64×64, TR = 2000 ms, TE = 30 ms, flip angle = 90°, number of slices = 32, slice thickness = 3 mm, and slice gap = 1.5 mm. Each condition, i.e., the ongoing condition and the PM condition was scanned in a separate session. The total fMRI scan had 126 dynamics.

### Data analysis

#### Behavioral data analysis

ANOVA in SPSS 16.0 was used to compare the difference between groups and conditions. Normality was checked before data analysis. All trials in PM condition were averaged for general reaction time and accuracy of PM task. Pearson correlation analysis was made between behavioral data (response time or accuracy) with image data (signal change percentage in fMRI, and parameters in DTI).

#### fMRI data analysis and statistics

The fMRI data were preprocessed and analyzed using the statistical parametric mapping package (SMP8, Wellcome Department of Cognitive Neurology, London). The first 6 dynamics were discarded. According to the standard preprocessing procedure, the individual fMRI data in two sessions was first realigned. Images of head movement over 4 mm within one session were excluded. The low-frequency temporal noise from the EPI was removed with a high-pass filter (1/128 Hz). T1 image was coregistered to the mean realigned image. The coregistered T1 image was segmented and its segment mat-file was used to normalize the individual EPI images. The normalized images were then spatially smoothed using a full-width half maximum (FWHM) three-dimensional Gaussian kernel of 10 mm. In first-level analysis, standard general linear model (GLM) was applied for modeling and inference of the statistical mapping to get contrasts between action and rest, i.e., between blocks of ongoing trials and rest in session 1, and between blocks of PM trials plus ongoing trials and rest in session 2. These contrast images in two sessions of each subject were entered in repeated measures ANOVA model for further analysis.

In group analysis, contrasts of each subject were entered into a flexible factorial analysis in SPM [47], [48]. The first factor was subject, and the second factor was group and the third was condition. To specify the contrasts, subjects and factors in the flexible design, the two contrasts of each subject were given a value of a 2*3 matrix. Comparisons were made between groups, conditions, and their interactions. PM-specific brain activation was resulted from contrast between the basic ongoing condition and PM condition in each group. The anatomical positions were labelled according to the MNI coordinates. To illustrate the anatomical connectivity for PM task, the generally activated brain network in three groups was saved as a region of interest (ROI) for further structural analysis (see below for details).

#### Tractography data analysis

The fiber tracking was performed with TrackVIS software [49], [50]. DTI data were first preprocessed by its Diffusion Toolkit. Individual gradient table was used for image orientation. Angle threshold was 42°, with the propagation algorithm set to FACT (fiber assignment by continuous tracking), as suggested in a previous tractography study on AD patients [51]. Fiber tracking was performed on native unwarped images to constrain mis-registration. Other details can be found in our previous tractography study [52].

**Figure 2:**
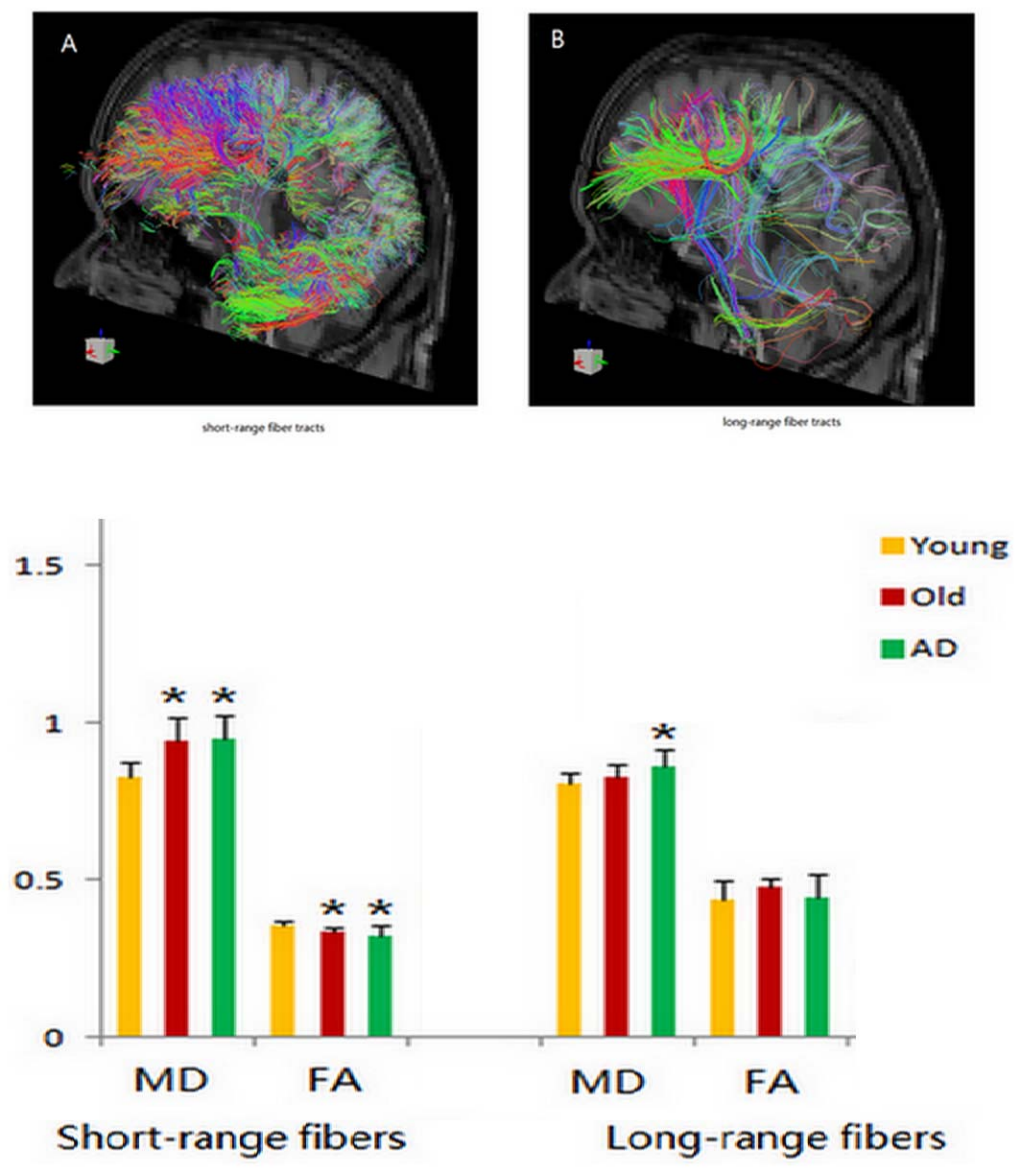
Tractography demonstrations of ROI from fMRI data for (A), short-range fiber tracts, and for (B), long-range fiber tracts. The lower part is the MD, FA of short-range fiber tracts, long-range fiber tracts in three groups. *, p<0.05, when compared to young group.

To compare fiber tracts supporting the PM task in three groups, the overall brain regions activated in PM condition of three groups were saved as a ROI. Conjunction group analysis in SPM8 (Global Null) was used [53], since the aim was to explore the structural connectivity of the brain network supporting PM task among the three groups, which had similar mental activities (Figure S1). A common ROI of PM condition was used since the basic PM related brain regions could represent all the involved brain regions. This could be more suitable for structural connectivity study to measure all the fiber tracts within the whole interactive network supporting the PM task. This common ROI was warped into each subject's DTI map to get an individual ROI. Only fibers with both endpoints in the ROI were calculated, as it was more relevant to the PM task. Fibers shorter than 4 mm were filtered from analysis [54]. The short-range fibers were identified with a maximum length of 35 mm, mainly cortiocortical fibers at regional or Brodmann scale [8]. Fibers longer than that were regarded as long-range fibers at a more global scale (Figure 2A, 2B for short-/long-range fibers). The fractional anisotrophy (FA), mean diffusivity (MD) of short-/long-range fibers within individual ROI were calculated separately. These DTI parameters were further analyzed to explore the white matter integrity. ANOVA in SPSS 16.0 was used to calculate the group means and their difference, with the significance level set at p<0.05.

## Results

### Behavioral results

Neuropsychological measurements (MMSE, digital span, verbal fluency, etc.) showed that older adults and AD patients had impaired working memory and frontal executive function, and slow information processing speed (Table 1). The healthy young and older subjects tended to perform the PM task equally well, while AD patients performed worse than the healthy young and older subjects. With regard to PM task reaction time, there was a trend that the healthy young adults performed faster than the healthy older adults, who performed faster than the AD patients. The detailed behavioral results of subjects in young adults, healthy older adults, and AD patients are shown in Table 2.

**Table 2:** The behavioral results in fMRI study.

	Young (13)	Old(12)	AD (13)
**ON.RT** (msec)	342±41	474±53	547±208*
**Onp.RT** (msec)	422±53	598±71*	720±196*
**PM.RT** (msec)	494±59	596±85	784±197* #
**PMall.RT** (msec)	440±51	598±71	742±206
**PMI.RT** (msec)	81±47	124±59	161±169
**ON.ACC** (100%)	99±01	99±02	96±04*
**Onp.ACC**(100%)	98±01	96±04	91±07* #
**PM.ACC** (100%)	97±04	97±05	91±13

The data are shown in Mean±SD. Numbers of subjects are shown in brackets. ON.RT, the average reaction time in ongoing condition; ONp.RT, the average reaction time of ongoing trials in PM condition; PM.RT the reaction time of PM trials in PM condition; PMall.RT, the overall reaction time in PM condition. PMI.RT, the PM interference score, calculate by Onp.RT-ON.RT; ON.ACC, the average accuracy in ongoing condition; ONp.ACC, the average accuracy of ongoing trials in PM condition; PM.ACC, the accuracy of PM trials in PM condition.

*, Group difference is significant when compared to healthy young adults, p<0.05;

# , Group difference is significant when compared to healthy older adults, p<0.05.

### fMRI study results

**Figure 3:**
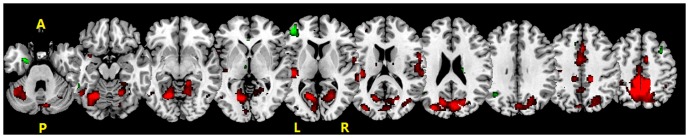
The difference of fMRI activity in PM conditions. Green color, between AD patients and healthy older adults, Red color, between healthy older adults and young adults. p<0.001, uncorrected. R, right side; L, left side.

**Figure 4:**
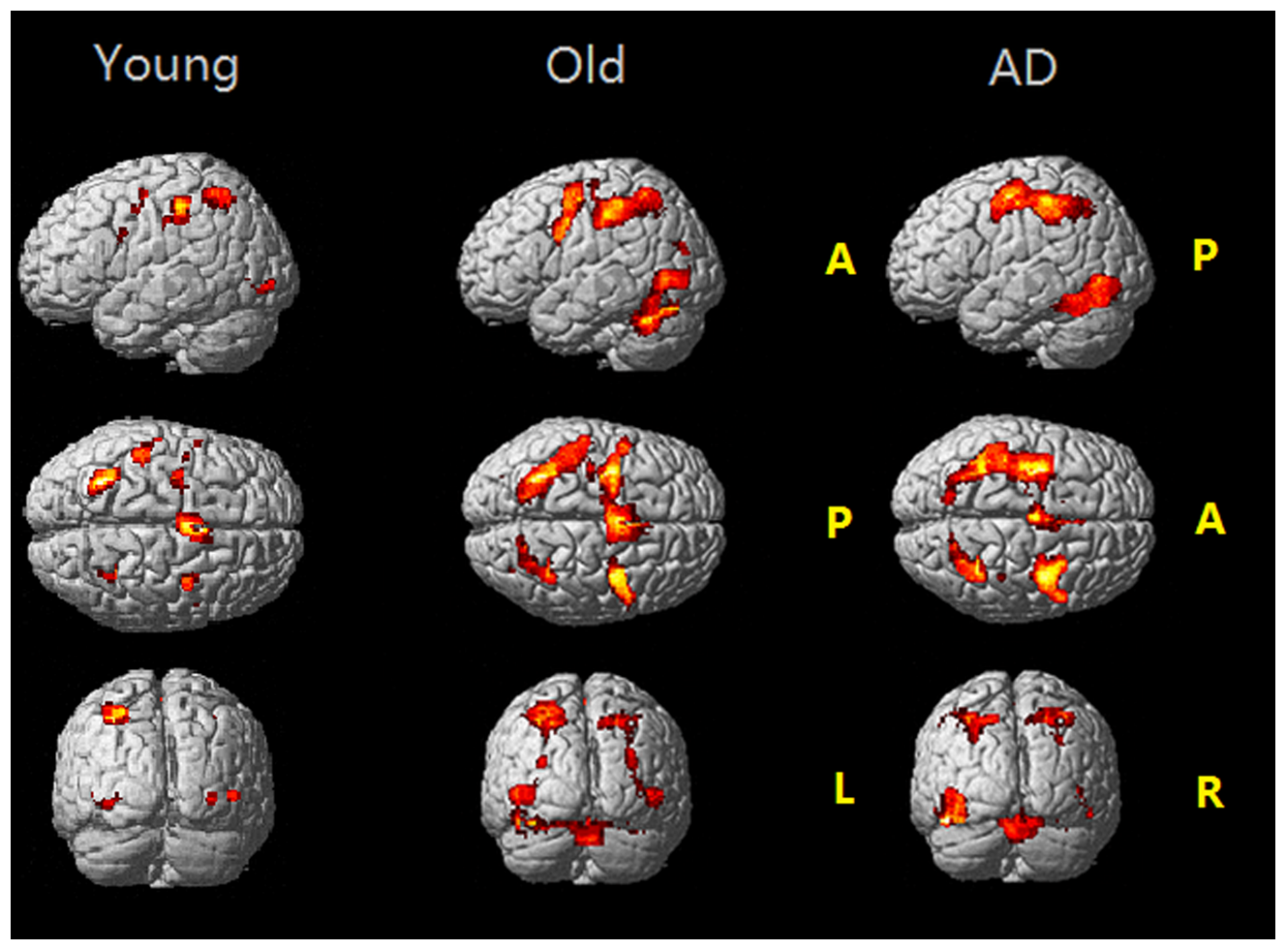
fMRI activity in PM conditions of the three groups: young adults, healthy older adults and AD patients. p<0.05, FWE corrected. R, right side; L, left side; A, anterior; P, posterior.

When comparing the general brain activations between the three groups, the young adults had much less brain activations than the other two groups, and AD patients had more anterior frontal lobe activation. In PM condition, the healthy older adults had dramatically increased brain activations, including bilateral supplementary motor area (SMA), the precentral gyrus, and inferior parietal lobe, bilateral cerebellum posterior lobe (declive), bilateral middle occipital gyri, bilateral thalamus, when compared to the young adults. AD patients had strong activations in PM condition similar to those of the healthy older adults, except more activation in the left inferior frontal gyrus (see Figure 3 and Table S1). Nonetheless, the patterns of brain activations were found. This indicated that core brain regions supporting the present PM task were similar in three groups (Figure 4). The SPM activity level was set at p<0.05, FWE corrected.

**Figure 5:**
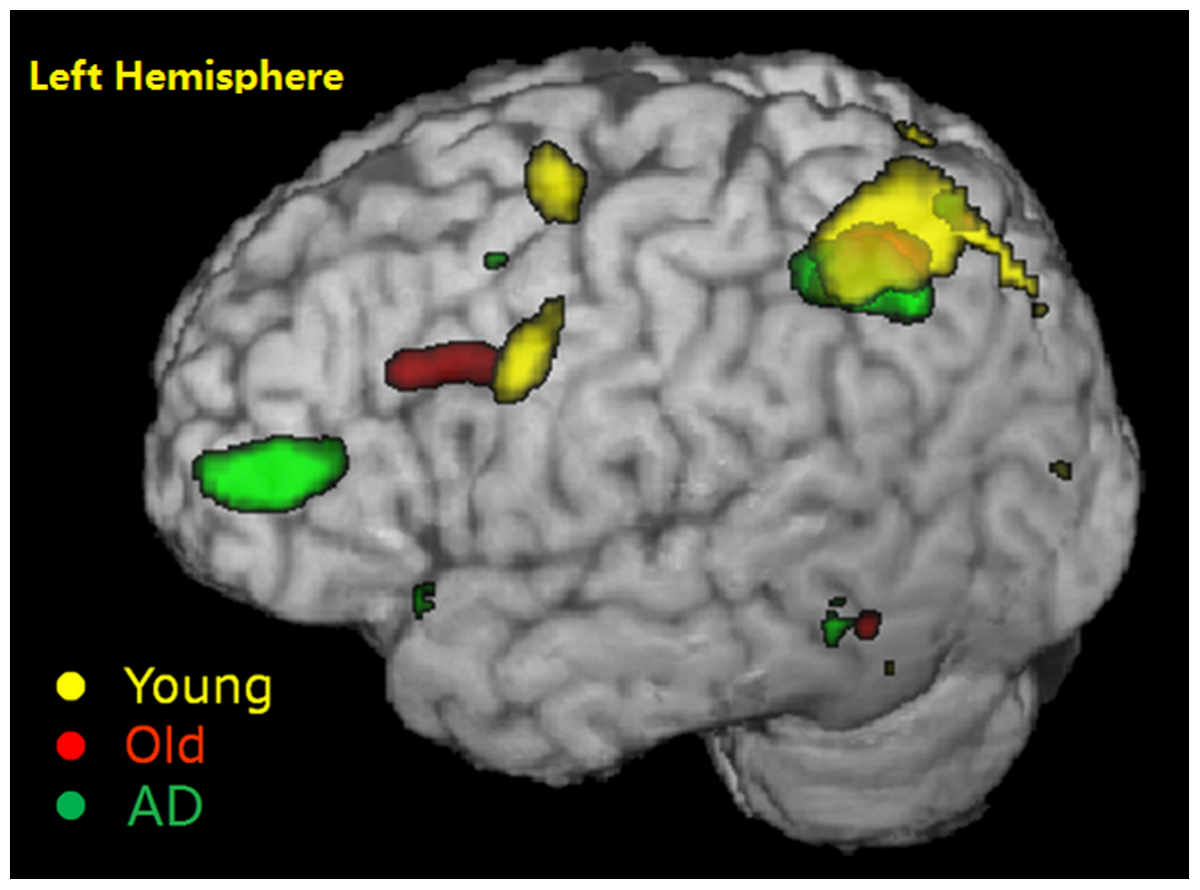
PM-specific brain regions in young adults (yellow), healthy older adults (red) and AD patients (green). p<0.001, uncorrected.

**Figure 6:**
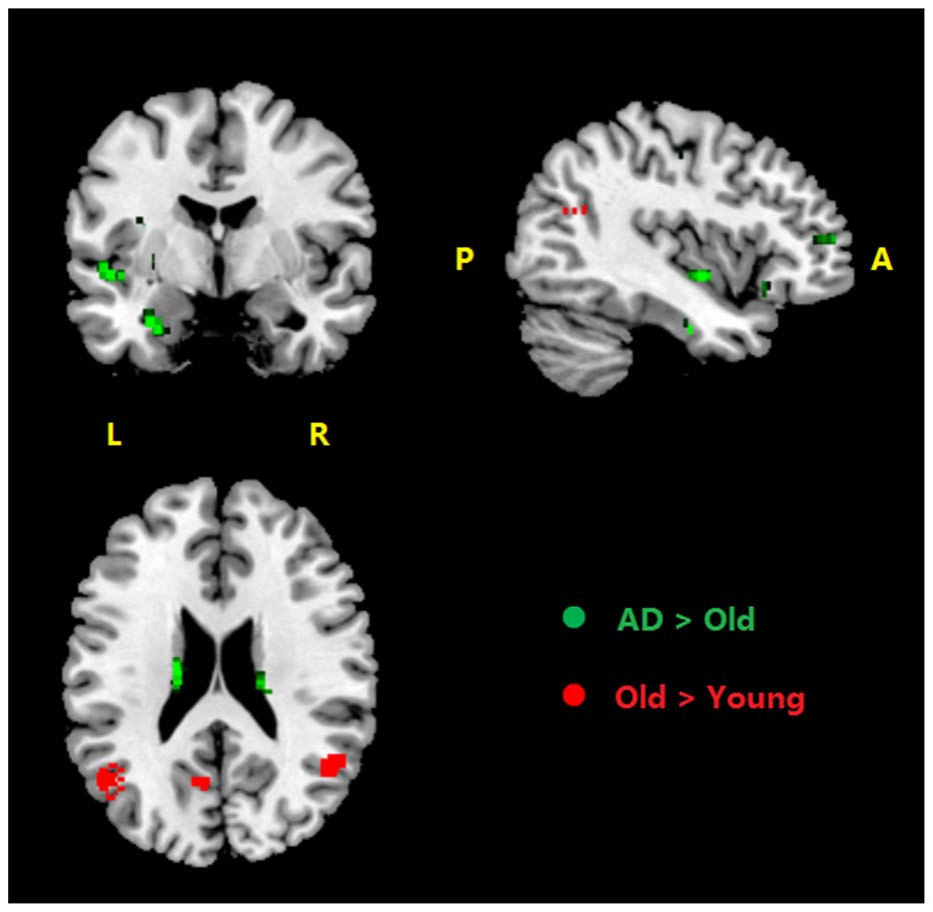
Difference of brain activations in PM-specific brain activations among the three groups. p<0.001 (uncorrected). Green areas represent regions where AD patients had more activity than healthy older adults; Red areas represent regions where healthy older adults had more activity than young adults. R, right side; L, left side; A, anterior; P, posterior.

The PM-specific brain regions were demonstrated by contrast between the brain activations in the ongoing condition and that in the PM conditions. Similar PM-specific brain regions were again found in each group, including the frontal lobe, bilateral SMA, the left fusiform gyrus, the left parietal lobe and right precuneus. However, the left frontal lobe activations were distinctly distributed in three groups. It was at the pars triangular region of the left inferior frontal lobe in AD patients, near the pars operculus regions in the healthy older adults, but at the dorsal part of premotor area in the young adults. These distributed areas roughly formed a frontal hierarchy along the rostral-caudal axis (see Figure 5 and Table S2). The activation level was set at p<0.001, uncorrected, for contrast between PM condition and ongoing condition. When compared directly among the groups by SPM, the AD patients still had high activation in the frontal activation, mainly the left frontal inferior orbitalis and pars triangular region than the healthy older adults (see Figure 6 and Table S3).

### Structural connectivity results

The white matter integrity within the PM activated brain network was compared between different groups to explore the effect of healthy and pathological aging on the structural connectivity relevant to PM task. When compared to the young adults, higher MD and lower FA of short-range fibers were found in both healthy older adults and AD patients; whereas only the MD of long-range fibers was significantly higher in the AD patients when compared to young adults (see lower part of Figure 2).

### Correlations between cognitive efficiency, functional activation and structural connectivity

**Figure 7:**
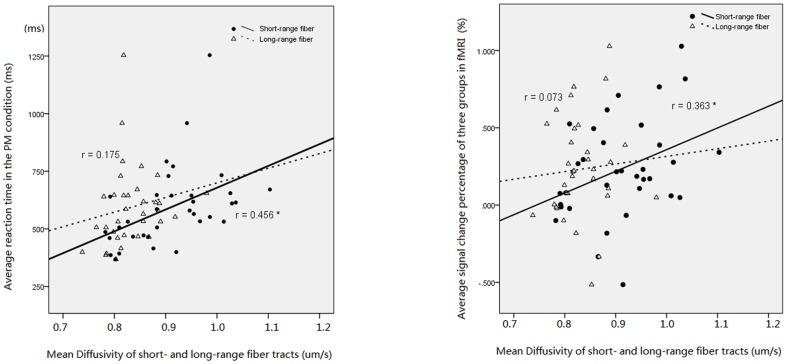
Correlation illustration: the left is correlations between MD of short-/long-range fiber tracts and average reaction time in PM condition; The right is the correlation between MD of short/long -range fiber tracts and fMRI signal change in PM condition among three groups. *, p<0.05. Short-/Long-range fibers, short- or long-range fiber tracts within the ROI of fMRI activations; MD, mean diffusivity (um/s); FA, fractional anisotrophy. *, Group difference is significant when compared to healthy young adults, p<0.05.

Correlation analysis among the three groups showed that the average response time in PM condition was correlated only with the MD of short-range fiber tracts (r = 0.456, p = 0.007), but not with the long-range fiber tracts (r = 0.175, p = 0.321). Also the overall fMRI signal change in brain activations of PM condition was correlated with the MD of short-range fiber tracts (r = 0.363, p = 0.035), but not with long-range fibers (r = 0.073, p  =  0.682). See Figure 7 for details.

## Discussion

This combined fMRI and tractography study investigated the role of structural connectivity in the effect of aging and AD on PM, an important cognitive task in daily living [22], [26]. The healthy older adults and mild AD patients could perform the adopted event-related PM task successfully in the MRI scanner. However, when compared to young adults, their efficiency to process this PM task was slower, accompanied by greater brain activations to compensate the reduced cognitive efficiency. These results are common seen in fMRI study on aging and dementia [55], [56].

### Frontal hierarchy in PM task

Despite the dramatically greater brain activations of the older groups in both ongoing condition and PM condition, the pattern of PM-specific brain activation was similar, and activation in the frontal lobe was observed in all three groups. Nonetheless, the positions were different and distributed along the rostrocaudal axis of the frontal lobe: The activation was at pars triangular region of the left inferior frontal lobe in AD patients, a more rostral region. In the healthy older and young adults, it was near the pars operculus regions and the dorsal part of premotor area, respectively. These are more caudal regions of the frontal lobe. This finding is extraordinary as PM-specific activation in the frontal lobe was found to vary only with different PM paradigms [57], while in the present study, a same PM task induced different frontal activation in subjects with different cognitive capacity. When directly comparing the frontal activation among the groups, the AD patients still had high activation in the left frontal inferior orbitalis and pars triangular region than the healthy older adults. These activations were in more rostral part of the frontal lobe. This distribution of frontal activation in three groups is in line with the cognitive hierarchy in the frontal lobe [58]–[60].

According to this assumption, the conflicts induced by cognitive task performance may be processed and organized hierarchically in the frontal lobe, especially the prefrontal cortex [58], [59]. A more rostral region responses to a cognitive task which is more uncertain or abstractive, whereas a more caudal region responses to more concrete task. The rostrocaudal axis in the prefrontal cortex may represent a hierarchical system of cognitive control [58], [61], which is important to PM task [62]. Thus, the rostral frontal activation of AD patients may imply that they could confront a more complex situation when reconfigurating the attention in PM task set, and the induced conflict may need a more abstractive strategy to deal with. On the other hand, more caudal frontal activations of the healthy young and older adults indicate that they had less uncertainty, and the performance might be more concrete for them and need less cognitive control during the PM task. This assumption can be partly examined by the behavioral data of increased PM interference effect on ongoing trial in AD patients. The interpolated PM trial made the subject constantly pay additional attention to them even when doing ongoing task and cause cognitive conflict [32]. The increased PM interference time in AD patient reflects their higher cognitive conflict than other groups when performing PM task. Together with fMRI data of hierarchical distribution of the frontal activation elicited, our results indicated that there were increasingly higher cognitive conflict and demand of better cognitive control of PM task in healthy older adults especially AD patients [63], [64]. The impaired PM task in AD may be attributed to their structural degeneration in key brain regions like the hippocampus, the frontal lobe, etc.. For example, PM impairment was found to correlate with stereotypical behaviors in frontotemporal dementia [65].

### Structural connectivity in brain network supporting PM task

The function of the network interacting is based on the structural connectivity. However, the integrity of white matter is especially vulnerable to the effect of aging and AD [66], [67]. FMRI data showed that although the frontal lobe plays a vital role in cognitive control, other brain regions were also found to support the PM task, including the precuneus (BA 7), the left inferior parietal lobe (BA 40), the thalamus, the supplementary motor area, etc. This is largely in line with previous fMRI studies on PM [24], [26], [28]. The success of PM task also needs basic visual, motor and relevant brain regions which altogether formed a network interacting with each other when performing PM task. Any disintegrity in this overall activated network could affect PM task performance, and it may attribute to the cognitive decline in healthy older adults [68] and clinical manifestations of AD [69], [70]. Given that the integrity of the white matter is vulnerable to the effect of aging and AD [66], [67], an overall active brain region in PM task was regarded as a ROI to explore the relevant structural connectivity impairment [65], [66],

Tractography results showed that the fiber tracts generally involved in PM task were impaired in the healthy older adults, and the impairment was more advanced in AD patients. A unique finding in the present study was that only the short-range fibers were vulnerable to aging effect, whereas the long-range fibers were more resistant. The short-range fibers are less myelinated, whereas the long-range fibers have thicker myelination which better insulates and protects the neuron and axon [7], [71]. In contrast, both short- and long-range fibers were impaired in AD patients. The pathological changes in AD could accelerate myelination degradation. Recent animal studies showed oligodendrocyte/myelin pathology in AD, and the accumulation of neurofibrillary tangles (NFT) may further disrupt the white matter connectivity [71]. These pathological changes may affect not only the less myelinated fibers, also the long-range fibers with thick myelination. These findings of impaired short-range fibers and long-range fibers may support the functional connectivity studies which reported both lower local clustering [10] and impaired global integrity in AD [72].

### Correlations between structural connectivity, functional activation and cognitive efficiency

The overall reaction time in PM condition was positively correlated with the mean diffusivity of the short-range fibers instead of the long-range fibers. This indicated that the information-processing efficiency in PM may be more related to the integrity of short-range fibers. It has important implication on the effort of isolating a central bottleneck of information processing, i.e., the bottleneck may posit in the local information processing rather than more global information integration which needs long-range fibers [73]. Since phase synchronization and neuronal synchrony is essential for conscious and cognitive processing [6], degradation of short-range fibers in aging and AD may lead to lower regional conduction velocity. This may delay the local high-frequency gamma band oscillation, and affect the information encoding efficiency [74].

The brain activation was also found to be more associated with the integrity of short-range fibers instead of long-range fibers. The more impairment in short-range fibers, the greater fMRI activation. Recent studies have revealed the coupling between fMRI activation and local electronic oscillation [74], [75], and cognitive performance can increase the coupling in brain regions engaged by the task [76]. The impairment of local connectivity in the older adults and AD patients may contribute to lower processing efficiency and correspondently, higher compensative neuronal activity. Similarly, a previous fMRI study on cognitive efficiency found that the slow performers have more frontal activation than the fast performers [77].

One limitation in this study was that DTI measure at sites of crossing-fibers may not be accurate as the sites with paralleling fibers. Diffusion spectrum diffusion may resolve the problem, while it generally takes a long time to scan and AD patient may not endure. The present DTI data have only one B0 image, so it may be susceptible to noise, whereas diffusion kurtosis image can be more sensitive to neural degeneration [78]. Another limitation is that the number of subjects in each group could have been higher. This was partly due to the considerable difficulty to recruit AD patients to successfully perform the PM task during the MRI scanning.

## Conclusion

In this combined fMRI and tractography study on aging and AD, we found that both healthy older adults and AD patients could perform the present PM paradigm successfully, but with lower cognitive efficiency and higher brain activations as compensation. Interestingly, the frontal activation of groups with different cognitive efficiency was arranged along the rostrocaudal-axis in the frontal lobe. This fMRI results together with increased PM interference in AD patients indicate that they may confront higher levels of conflict and need different strategies during PM task performance. Structural connectivity data demonstrated that the cognitive efficiency of the PM task performance among groups was more related to the disintegrity of short-range fibers. The latter was also correlated with the increased brain activation. It is suggested that the disintegrity of short-range fibers or local integrity may contribute to the lower cognitive efficiency and higher compensatory brain activation in healthy older adults and more in AD patients.

## Supporting Information

Figure S1 **Region of Interest resulted from fMRI activation in PM condition.**
(TIF)

Table S1 **Differences in PM conditions.** L, represents left; R, represent right. p<0.001, uncorrected; at least 10 voxels.
(DOCX)

Table S2 **PM-specific activation in three groups.** L, represents left; R, represent right. p<0.001, uncorrected; at least 10 voxels.
(DOCX)

Table S3 **Difference in PM-specific brain activation in three groups.** L, represents left; R, represent right. p<0.001, uncorrected; at least 10 voxels.
(DOCX)
