# Supplementary figures and images for: The Relevance of Short-Range Fibers to Cognitive Efficiency and Brain Activation in Aging and Dementia
Source: PLoS One. 2014 Apr 2;9(4):e90307. doi: 10.1371/journal.pone.0090307 (PMC3973665; doi:10.1371/journal.pone.0090307)

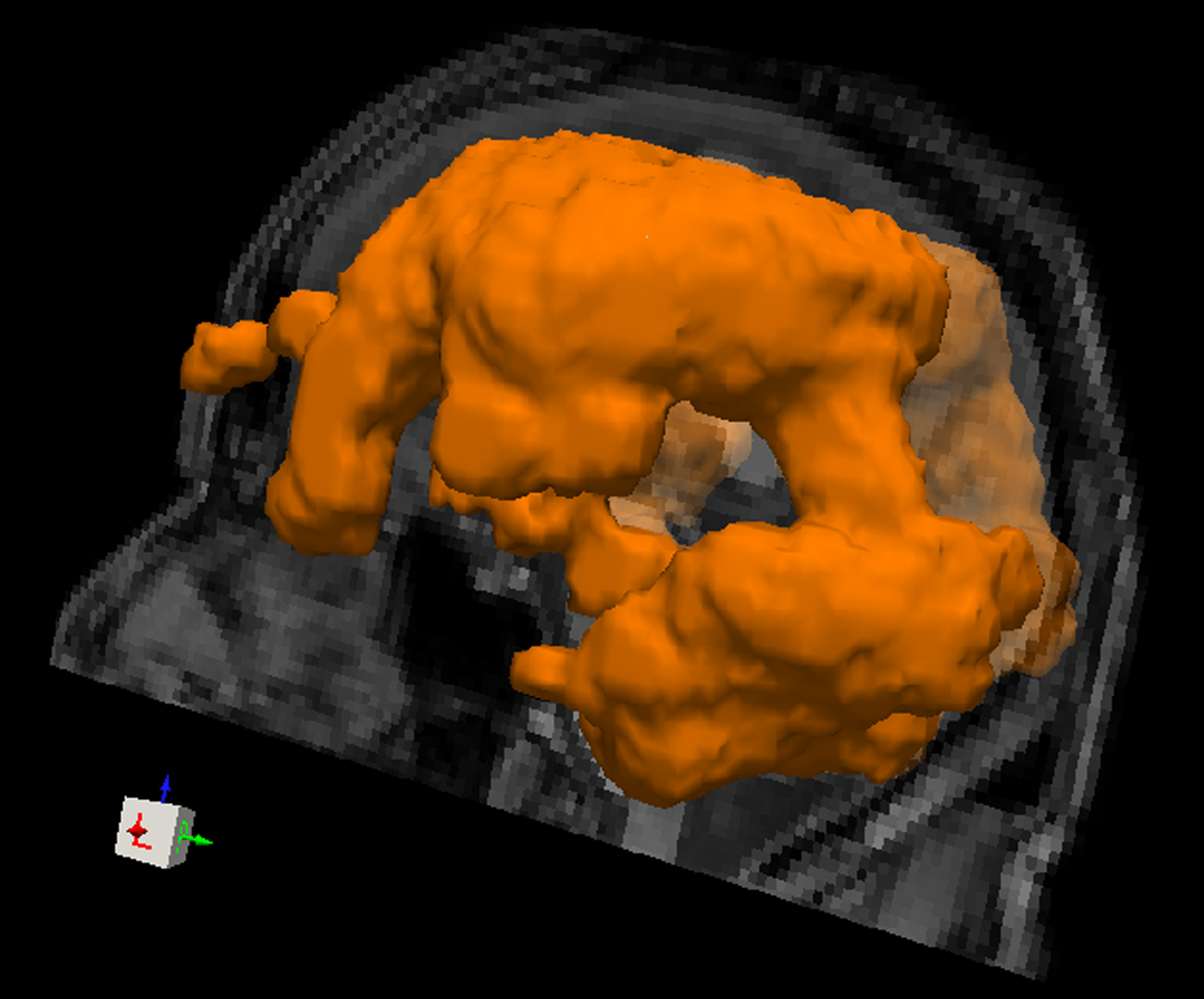

Supplement: Figure S1 — Region of Interest resulted from fMRI activation in PM condition. (TIF) [file pone.0090307.s001.tif]
