# Supplementary material for: The Relevance of Short-Range Fibers to Cognitive Efficiency and Brain Activation in Aging and Dementia
Source: PLoS One. 2014 Apr 2;9(4):e90307. doi: 10.1371/journal.pone.0090307 (PMC3973665; doi:10.1371/journal.pone.0090307)
Supplement: Table S1 — Differences in PM conditions. L, represents left; R, represent right. p<0.001, uncorrected; at least 10 voxels. (DOCX) [file pone.0090307.s002.docx]

Table S1: Differences in PM conditions

| Anatomic region |  | Voxels |  | x | y | z |  | t-val |
| --- | --- | --- | --- | --- | --- | --- | --- | --- |
| *Healthy Older Adults – Young adults* |  |  |  |  |  |  |  |  |
| L Precuneus area |  | 3095 |  | -10 | -56 | 52 |  | 5.48 |
| R Precuneus area |  | 2750 |  | 10 | -54 | 50 |  | 5.54 |
| L Precuneus area |  | 1158 |  | -12 | -52 | 68 |  | 4.48 |
| L Cerebelum |  | 1108 |  | -30 | -64 | -22 |  | 5.17 |
| L Lingual Gyrus |  | 1027 |  | -20 | -62 | -12 |  | 4.50 |
| L Middle Occipital Gyrus |  | 379 |  | -32 | -82 | 18 |  | 4.19 |
| L Superior Temporal Gyrus |  | 233 |  | -64 | -26 | 16 |  | 4.45 |
| L Heschl |  | 108 |  | -40 | -26 | 12 |  | 4.01 |
| R Supplementary Motor Area |  | 94 |  | 10 | -4 | 54 |  | 3.69 |
| R Middle Cingulum Gyrus |  | 62 |  | 4 | 18 | 42 |  | 4.46 |
| R Cerebelum |  | 41 |  | 18 | -56 | -14 |  | 4.11 |
| R Cerebelum Crus ii |  | 38 |  | 10 | -76 | -32 |  | 3.79 |
| R Lingual Gyrus |  | 37 |  | 16 | -42 | -12 |  | 3.52 |
|  |  |  |  |  |  |  |  |  |
| *AD patients – Healthy Older Adults* |  |  |  |  |  |  |  |  |
| L Inferior Frontal Triangularis |  | 168 |  | -40 | 44 | 6 |  | 4.24 |
| L Angular Gyrus |  | 64 |  | -38 | -62 | 36 |  | 3.86 |
| L ParaHippocampal area |  | 63 |  | -28 | -2 | -30 |  | 4.06 |
| R Middle Frontal Gyrus |  | 60 |  | 32 | 10 | 54 |  | 3.77 |
| L Inferior Temporal Gyrus |  | 46 |  | -56 | -48 | -12 |  | 4.26 |
| R Caudate Nucleus |  | 27 |  | 20 | -22 | 22 |  | 4.04 |
| L Superior Medial Frontal Gyrus | | 13 |  | -8 | 26 | 42 |  | 3.82 |

L, represents left; R, represent right. p < 0.001, uncorrected; at least 10 voxels.
