# Supplementary material for: The Relevance of Short-Range Fibers to Cognitive Efficiency and Brain Activation in Aging and Dementia
Source: PLoS One. 2014 Apr 2;9(4):e90307. doi: 10.1371/journal.pone.0090307 (PMC3973665; doi:10.1371/journal.pone.0090307)
Supplement: Table S2 — PM-specific activation in three groups. L, represents left; R, represent right. p<0.001, uncorrected; at least 10 voxels. (DOCX) [file pone.0090307.s003.docx]

Table S2. PM-specific activation in three groups.

| Anatomic region |  | |  | | Voxels | |  | | x | | y | | z | |  | | t-val | |  |
| --- | --- | --- | --- | --- | --- | --- | --- | --- | --- | --- | --- | --- | --- | --- | --- | --- | --- | --- | --- |
| *Young adults* |  | |  | |  | |  | |  | |  | |  | |  | |  | |  |
| R Supplementary Motor Area |  | |  | | 391 | |  | | 8 | | 10 | | 50 | |  | | 5.46 | |  |
| R PreMotor Area |  | |  | | 192 | |  | | 48 | | 6 | | 36 | |  | | 5.24 | |  |
| L Premotor Area |  | |  | | 172 | |  | | -42 | | 2 | | 32 | |  | | 4.60 | |  |
| L Superior Frontal Gyrus |  | |  | | 116 | |  | | -22 | | -4 | | 56 | |  | | 4.37 | |  |
| R Superior Frontal Gyrus |  | |  | | 67 | |  | | 20 | | 6 | | 52 | |  | | 3.92 | |  |
| L Inferior Parietal Gyrus |  | |  | | 1011 | |  | | -28 | | -56 | | 44 | |  | | 6.16 | |  |
| R Precuneus |  | |  | | 587 | |  | | 12 | | -66 | | 42 | |  | | 6.14 | |  |
| L Fusiform Gyrus |  | |  | | 11 | |  | | -30 | | -74 | | -14 | |  | | 3.52 | |  |
| R Fusiform Gyrus |  | |  | | 41 | |  | | 32 | | -60 | | -14 | |  | | 3.62 | |  |
| R Middle Occipital Gyrus |  | |  | | 179 | |  | | 32 | | -80 | | 10 | |  | | 4.47 | |  |
| L Middle Occipital Gyrus |  | |  | | 43 | |  | | -22 | | -88 | | 8 | |  | | 3.53 | |  |
| L Anterior Cerebelum Lobe |  | |  | | 81 | |  | | -36 | | -58 | | -28 | |  | | 3.79 | |  |
| L Cerebelum Declive |  | |  | | 114 | |  | | -8 | | -78 | | -14 | |  | | 3.87 | |  |
|  |  | |  | |  | |  | |  | |  | |  | |  | |  | |  |
| *Healthy Older Adults* |  | |  | |  | |  | |  | |  | |  | |  | |  | |  |
| R Inferior Frontal Gyrus |  | |  | | 53 | |  | | 38 | | 20 | | 28 | |  | | 3.79 | |  |
| L Inferior Frontal Gyrus |  | |  | | 102 | |  | | -44 | | 22 | | 30 | |  | | 3.75 | |  |
| L Angular Gyrus |  | |  | | 624 | |  | | -30 | | -48 | | 36 | |  | | 4.18 | |  |
| R Precuneus |  | |  | | 864 | |  | | 10 | | -66 | | 44 | |  | | 5.60 | |  |
| L Fusiform Gyrus |  | |  | | 43 | |  | | -46 | | -54 | | -14 | |  | | 3.79 | |  |
| R Insula |  | |  | | 13 | |  | | -42 | | 8 | | 12 | |  | | 3.70 | |  |
| L Hippocampus |  | |  | | 17 | |  | | -26 | | -36 | | 10 | |  | | 3.94 | |  |
|  |  | |  | |  | |  | |  | |  | |  | |  | |  | |  |
| *AD Patients* | |  | |  | |  | |  | |  | |  | |  | |  | |  | |
| L Middle Frontal Gyrus |  | |  | | 314 | |  | | -38 | | 46 | | 10 | |  | | 4.75 | |  |
| L Insula |  | |  | | 75 | |  | | -38 | | 14 | | -14 | |  | | 3.86 | |  |
| R Inferior Frontal Gyrus |  | |  | | 159 | |  | | 52 | | 12 | | 34 | |  | | 3.80 | |  |
| L Angular Gyrus |  | |  | | 598 | |  | | -32 | | -58 | | 38 | |  | | 5.19 | |  |
| R Parietal Inferior Lobe |  | |  | | 690 | |  | | 36 | | -52 | | 50 | |  | | 4.17 | |  |
| R Precuneus |  | |  | | 332 | |  | | 8 | | -64 | | 48 | |  | | 4.47 | |  |
| R Fusiform Gyrus |  | |  | | 77 | |  | | 40 | | -58 | | -12 | |  | | 4.08 | |  |
| R Superior Temporal Gyrus |  | |  | | 22 | |  | | 36 | | 4 | | -24 | |  | | 3.68 | |  |
| L Inferior Temporal Gyrus |  | |  | | 126 | |  | | -50 | | -48 | | -12 | |  | | 4.08 | |  |
| R Lentiform Nucleus |  | |  | | 166 | |  | | 22 | | 6 | | 4 | |  | | 4.47 | |  |
| R Caudate |  | |  | | 28 | |  | | 16 | | -18 | | 20 | |  | | 4.01 | |  |
| L Caudate |  | |  | | 60 | |  | | -18 | | -10 | | 24 | |  | | 4.31 | |  |
| R Hippocampus |  | |  | | 19 | |  | | 40 | | -28 | | -14 | |  | | 3.77 | |  |
| L Thalamus |  | |  | | 50 | |  | | -2 | | -26 | | -4 | |  | | 3.68 | |  |
| L Anterior Cerebelum Lobe |  | |  | | 11 | |  | | -22 | | -40 | | -28 | |  | | 3.39 | |  |

L, represents left; R, represent right. p < 0.001, uncorrected; at least 10 voxels.
