# Supplementary material for: The Relevance of Short-Range Fibers to Cognitive Efficiency and Brain Activation in Aging and Dementia
Source: PLoS One. 2014 Apr 2;9(4):e90307. doi: 10.1371/journal.pone.0090307 (PMC3973665; doi:10.1371/journal.pone.0090307)
Supplement: Table S3 — Difference in PM-specific brain activation in three groups. L, represents left; R, represent right. p<0.001, uncorrected; at least 10 voxels. (DOCX) [file pone.0090307.s004.docx]

Table S3. Difference in PM-specific brain activation in three groups.

| Anatomic region |  | Voxels |  | x | y | Z |  | t-val |
| --- | --- | --- | --- | --- | --- | --- | --- | --- |
| *Healthy Older Adults - Young adults* |  |  |  |  |  |  |  |  |
| L Middle Cingulum Area |  | 108 |  | 0 | -40 | 38 |  | 3.81 |
| R Angular Gyrus |  | 52 |  | 48 | -56 | 24 |  | 3.60 |
| L Middle Temporal Gyrus |  | 15 |  | -50 | -64 | 22 |  | 3.48 |
|  |  |  |  |  |  |  |  |  |
| *AD patients – Healthy Older Adults* |  |  |  |  |  |  |  |  |
| L Temporal Superior Gyrus |  | 230 |  | -48 | -12 | -2 |  | 4.74 |
| L Temporal Inferior Gyrus |  | 14 |  | -38 | -10 | -28 |  | 4.09 |
| L Amygdala |  | 26 |  | -30 | -2 | -26 |  | 4.02 |
| R Caudate |  | 21 |  | 20 | -20 | 20 |  | 3.97 |
| L Caudate |  | 40 |  | -18 | -12 | 24 |  | 3.90 |
| L Frontal Inferior Orbitalis |  | 45 |  | -38 | 18 | -14 |  | 3.54 |
| L Frontal Superior Gyrus |  | 14 |  | -22 | 8 | 44 |  | 3.51 |
| L Frontal Inferior Triangularis |  | 14 |  | -44 | 46 | 10 |  | 3.50 |

L, represents left; R, represent right. p < 0.001, uncorrected; at least 10 voxels.
